# Experimental infection of cattle with ovine *Dichelobacter nodosus* isolates

**DOI:** 10.1186/s13028-015-0148-5

**Published:** 2015-09-25

**Authors:** Maren Knappe-Poindecker, Hannah Joan Jørgensen, Tim Kåre Jensen, Bereket Tesfamichael, Martha Jakobsen Ulvund, Lisbeth Hektoen, Terje Fjeldaas

**Affiliations:** Norwegian University of Life Sciences, PO Box 8146, 0033 Oslo, Norway; Norwegian Veterinary Institute, PO Box 750, 0106 Oslo, Norway; National Veterinary Institute, Technical University of Denmark, Bülowsvej 27, 1870 Frederiksberg C, Denmark; Norwegian University of Life Sciences, Campus Sandnes, Kyrkjevegen 332/334, 4325 Sandnes, Norway; Norwegian Sheep Health Service, Animalia, PO Box 396, Økern, 0513 Oslo, Norway

**Keywords:** *Dichelobacter nodosus*, Interdigital dermatitis, Cross-infection, Sheep, Cattle

## Abstract

**Background:**

*Dichelobacter nodosus* is the main causative agent of ovine footrot, and there are strong indications that the bacterium can be transferred to cattle grazing on the same pasture as sheep. The aim of this study was to investigate if benign and virulent *D. nodosus* strains isolated from sheep can be transferred to the interdigital skin of cattle under experimental conditions. Further, we wanted to observe the impact of such infection on bovine foot health, and test the effect of topical chlortetracycline (Cyclo spray^®^: Eurovet) on the infection.

**Findings:**

Six heifers were included in the study. After an initial 18-day maceration period, three heifers were inoculated on one single foot with a benign strain and three with a virulent strain by adding bacterial suspension in a bandage. The bandages were left on for 17 days, and when removed, *D. nodosus* was isolated from all six heifers. All six heifers developed interdigital dermatitis. In five of the heifers *D. nodosus* organisms were demonstrated within the epidermis. Twenty-four days after treatment with chlortetracycline all heifers were negative by cultivation, but tested positive for *D. nodosus* by polymerase chain reaction (PCR). Two of the six heifers still tested positive for *D. nodosus* by PCR 49 days after treatment. After 70 days, all heifers tested negative for *D. nodosus*.

**Conclusions:**

This study shows that both virulent and benign *D. nodosus* strains originating from sheep can be transferred to naïve heifers under experimental conditions. Further, the study supports the hypothesis that infections with virulent *D. nodosus* in cattle are associated with interdigital dermatitis. No conclusion regarding the treatment of *D. nodosus* infection with chlortetracycline was possible.

## Findings

*Dichelobacter nodosus*, a Gram-negative anaerobic bacterium, is the main causative agent of ovine footrot [1]. In cattle, *D. nodosus* is associated with interdigital and digital dermatitis, but the bacterium is also commonly present on the digital skin of healthy cows [2, 3]. The bacterium produces extracellular proteases believed to play a role in its pathogenicity. The proteases differ with respect to thermostability, as tested in the gelatin gel (GG) test [4]. In sheep, strains producing thermostable proteases are considered more virulent than strains producing thermolabile proteases [5]. Virulence testing is not performed routinely on *D. nodosus* isolates retrieved from cattle, but a previous study has shown that isolates from cattle without contact with sheep were all defined as benign by the GG-test [2].

In Norway, sheep and cattle frequently graze on the same pasture. In 2008, when ovine footrot was diagnosed in Norway for the first time in 60 years, concerns were raised whether cross-infection between sheep and cattle possibly could occur [6]. Previous research has indicated that cross-infections of both virulent and benign strains between sheep and cattle have occurred on pasture [7–9]. However, it has been suggested that cattle do not represent an important reservoir of virulent *D. nodosus* strains [1].

The aim of this study was to investigate if benign and virulent *D. nodosus* isolated from sheep could colonize the interdigital skin of cattle under experimental conditions. Further, we wanted to observe the impact of the infection on bovine foot health, and to test the effect of topical chlortetracycline (Cyclo spray^®^: Eurovet) on the infection.

The trial was conducted at the Norwegian University of Life Sciences, Campus Sandnes, and included six Norwegian Red heifers approximately 16-months old, purchased from two different commercial dairy farms. To ascertain that the heifers were negative for *D. nodosus*, sterile cotton swabs for polymerase chain reaction (PCR) analysis were collected 2 months prior to the trial and analysed as described in Knappe-Poindecker et al. [10]. After bacterial sampling and until the end of the trail, the heifers were kept in a pen without contact with other animals. The study protocol was approved by the National Animal Research Authority prior to the start of the trial (protocol number 3554).

**Fig. 1:**
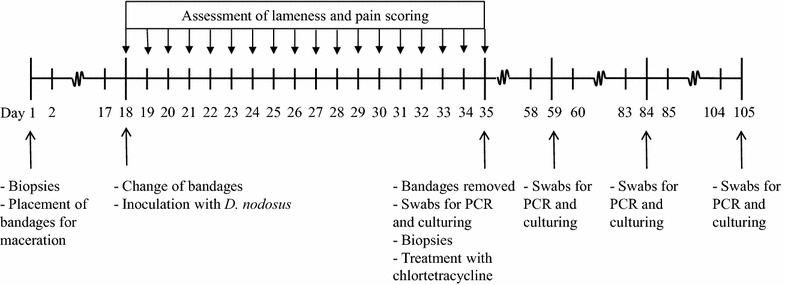
Timeline for the infection trail of six naïve heifers with *Dichelobacter nodosus*

The timeline for the trial is illustrated in Fig. 1. Four days before the start of the trial, the heifers were moved to an empty tie stall where they were housed throughout the trial. For practical reasons, the right front foot was chosen for inoculation. On day 1 of the trial, the heifers were sedated with Xylazine 20 mg/ml (Narcoxyl; MSD Animal Health). While lying down, the foot health was recorded and skin biopsies were taken with a 3 mm biopsy punch (Miltex, Inc., USA).

The biopsies were fixed in 10 % neutral buffered formalin, processed by routine methods, paraffin embedded and sectioned at 4 µm. Tissue sections were stained by haematoxylin and eosin. Additionally, sections were subjected to fluorescence in situ hybridization (FISH) using oligonucleotide probes targeting 16S ribosomal RNA of *D. nodosus* (5′ cat gca ccg ttc ttc act 3′), and *Treponema* spp (5′ cag aaa cyc gcc ttc gcc 3′), and domain bacterium (Eub 338) as previously described [3]. The oligonucleotide probes were 5′ labelled with fluorescein isothiocyanate or Cy3 and hybridization was carried out at 46 °C. An Axioimager M1 epifluorescence microscope equipped for epifluorescence with a 100-W HBO lamp and filter sets 43 and 38 were used to visualize Cy3 and FITC, respectively. Images were obtained using an AxioCam MRm version 3 FireWiremonocrome camera and AxioVision software, version 4.5 (Carl Zeiss, Oberkochen, Germany).

The foot was bandaged and 10 ml of tap water was added to create moist conditions [11]. The distal part of the foot was covered by canvas for protection and was then secured with an adhesive bandage. The position of the bandages were inspected daily and the heifers were also daily assessed for lameness by locomotion scoring or signs of pain [12].

On day 18, the heifers were sedated as described above and the bandages were removed. After inspection of the digits, the bandages were replaced and 20 ml bacterial suspensions containing 10^6^–10^7^ bacteria/ml prepared as described in [10] were added to the bandages. All heifers were exposed to *D. nodosus* originating from sheep. Three of the heifers were exposed to a *D. nodosus* strain defined as benign by the GG-test, belonging to serogroup G. Even though the strain was benign, some of the sheep in the flock from which it was isolated had slightly under-running lesions. The other three heifers were infected with a virulent strain belonging to serogroup A.

On day 35, the bandages were removed, the digits were inspected, swabs for culturing were collected from the interdigital skin, placed in Transystem Amies agar gel medium with charcoal (Copan, Brescia, Italy) and processed as described in [10]. Also swabs for PCR were collected and additional skin biopsies were taken and processed as described above. Afterwards, the heifers were treated topically with chlortetracycline spray once (Cyclo spray^®^: Eurovet). Additional swabs for cultivation and PCR were taken on days 59, 84 and 105 of the trial. For PCR-analysis, DNA was extracted from the swabs in PBS with EDTA using a nucliSENS easyMAG extractor (bioMèrieux, Boxtel, The Netherlands) following the manufacturer’s instructions. DNA from cultured isolates was obtained by diluting broth culture 1:5 in double distilled water followed by boiling for 1 min. Extracted DNA was stored at −20 °C. *D. nodosus* was detected using a real-time PCR as described previously [13].

The heifers had clinically healthy digits and tested negative for *D. nodosus* by PCR 2 months prior to the trial. Histopathology of the skin biopsies on day 1 showed normal skin morphology and no bacteria, including *D. nodosus,* was detected by FISH.

When the bandages were removed on day 35 of the trial the foot health was inspected and found normal. *D. nodosus* was isolated by culturing and detected by PCR from the surface of the interdigital skin of all heifers. The three heifers infected with the benign strain had developed interdigital dermatitis in the interdigital space, and in two of these heifers dermal lesions were also present at the dorsal part of the coronary band. The heifers infected with the virulent strain all had slightly milder lesions which resembled mild interdigital dermatitis. None of the heifers were found lame by locomotion scoring during the trial.

**Fig. 2:**
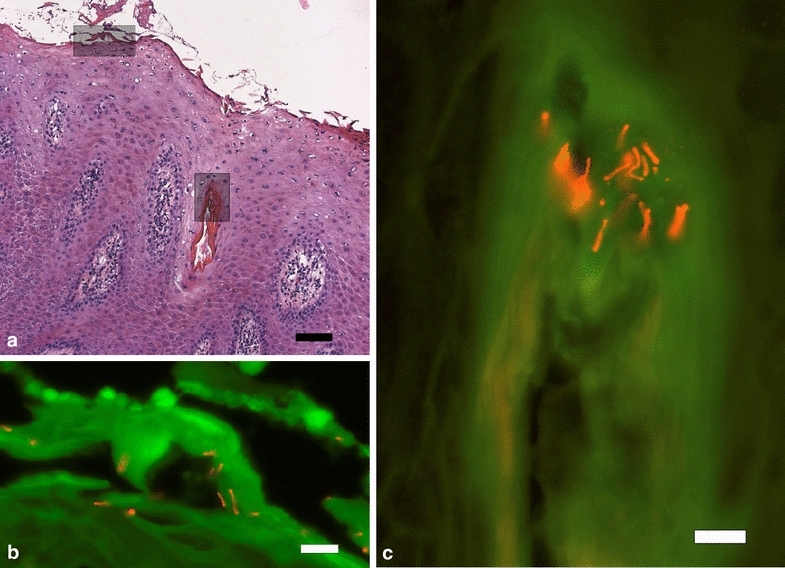
Epidermis from the foot of a heifer experimentally challenged with a benign *D. nodosus* strain. **a** showing increased thickness of the epithelial layers (acanthosis) and mal-keratinization, H&E staining, bar 100 µm. Inserted, demonstration of *D. nodosus* by fluorescent in situ hybridization in **b** within dyskeratinized epidermis and **c** a hair follicle. Demonstration of *D. nodosus* by Cy3 labelled oligonucleotide probe, *bars* 10 µm

The skin biopsies from all three heifers infected with the benign strain and the skin biopsies in two of the heifers infected with the virulent strain showed mild to moderate epidermal changes characterized by acanthosis as well as degeneration and mal-keratinization (large epithelial cells without keratin formation and with persistent nuclei in stratum corneum) or loss of the stratum corneum. The biopsy from the third heifer infected with the virulent strain, was found negative for bacteria and the epidermis had normal histomorphology. FISH identified multiple *D. nodosus* organisms, as the only bacterium, invading the superficial epidermal layers of all three heifers infected with the benign strain and one heifer infected with the virulent strain. The *D. nodosus* organisms also invaded hair follicles (Fig. 2).

On day 59, 24 days after treatment with chlortetracycline, all heifers were negative for *D. nodosus* by cultivation, but all were positive by PCR. On day 84, only two heifers, both infected with the benign strain, tested positive by PCR. On day 105, all six heifers tested negative.

This study shows that both benign and virulent ovine *D. nodosus* strains can colonize the interdigital skin and induce lesions in cattle under experimental conditions. The findings are in agreement with a recent study which indicated that cross-infection of *D. nodosus* can occur and is of importance to the on-going Norwegian elimination programme of ovine footrot [7, 14].

All heifers in this study developed interdigital dermatitis, but the heifers infected with the virulent strain developed milder gross lesions and histopathological alterations than the heifers infected with the benign strain. In previous studies, virulent *D. nodosus* in cattle has been associated with interdigital dermatitis indistinguishable from infections caused by benign strains [7, 15]. Even though the differences in symptoms and histology observed between our two study groups were distinct, the number of animals was too few to evaluate the significance of this finding.

The association between *D. nodosus* and lameness in sheep is well known [16], but in cattle this association has not been completely elucidated. Bennett et al. [17] considered it possible that the presence of the bacterium on the feet of lame cattle could be of significance for lameness. However, interdigital dermatitis, which is associated with *D. nodosus*, does normally not cause lameness in cattle [2, 18], and the absence of lameness in the six heifers included in our study agrees with this previous finding and supports the hypothesis that the presence of *D. nodosus* in cattle is not necessarily associated with lameness.

The effect of chlortetracycline on infections with *D. nodosus* is not proven. Even though all heifers were negative by cultivation, all of them tested positive by PCR on days 59, 24 days after treatment with chlortetracycline. Two of the heifers, both infected with the benign strain, also tested positive by PCR on day 84, i.e. 49 days after treatment. However, even if the treatment was effective, the heifers were expected to test positive by PCR for some time after treatment because PCR detects both living and dead bacteria [19].

Even though cattle have been claimed not to be reservoirs for virulent *D. nodosus*, cattle infected with *D. nodosus* on pasture have remained infected for at least 8 months [7, 9]. Also in the present study, *D. nodosus* organisms were found invading the epidermis, including the hair follicles, and benign strains were detected by PCR for up to 7 weeks, and virulent strains two and a half weeks after bandage removal. The bandage removal, and the fact that a front foot was chosen for inoculation instead of a hind foot, probably implicated drier, suboptimal conditions for *D. nodosus*, which may have reduced the time the bacterium persisted [20]. Detection of dead bacteria for several weeks on skin where the surface is continually renewed is unlikely and our results thus support the aforementioned studies suggesting that *D. nodosus* may persist in cattle.

In conclusion, this study shows that both benign and virulent *D. nodosus* originating from sheep can colonize the interdigital skin of naïve heifers under experimental conditions. Further, the study supports the hypothesis that infections with virulent *D. nodosus* in cattle are associated with interdigital dermatitis. No conclusion regarding the treatment of *D. nodosus* infection with chlortetracycline was possible. The ability of *D. nodosus* to cross between sheep and cattle is epidemiologically important, and cattle should be considered a possible source of virulent *D. nodosus* to sheep when planning and implementing elimination programs.
